# Management Strategies and Outcomes for Small Intestinal Neuroendocrine Tumours with Involvement of the Superior Mesenteric Vessels: A Systematic Review

**DOI:** 10.3390/curroncol30100664

**Published:** 2023-10-18

**Authors:** Elizabeth Kmiotek, Sakina Lakda, Aditya Borakati, Olagunju Ogunbiyi, Dalvinder Mandair, Martyn Caplin, Christos Toumpanakis, Reza Mirnezami

**Affiliations:** 1 Department of Colorectal Surgery, The Royal Free Hospital, Pond Street, Hampstead, London NW3 2QG, UKolagunju.ogunbiyi@nhs.net (O.O.); 2 University College London Medical School, 74 Huntley Street, London WC1E 6DE, UK; sakina.lakda.19@ucl.ac.uk; 3 Division of Surgery and Interventional Science, University College London, Royal Free Hospital Campus, 9th Floor, Pond Street, Hampstead, London NW3 2QG, UK; aditya.borakati@nhs.net; 4 Neuroendocrine Tumour Unit, The Royal Free Hospital, Pond Street, Hampstead, London NW3 2QG, UK; dalvinder.mandair@nhs.net (D.M.); martyn.caplin@nhs.net (M.C.); c.toumpanakis@nhs.net (C.T.)

**Keywords:** small intestine, neuroendocrine tumours, carcinoid tumour, neuroendocrine carcinoma, mesentery

## Abstract

Small intestinal neuroendocrine tumours (SI-NETs) are the most common small intestinal tumours. A particularly challenging subset of these tumours is those that involve the superior mesenteric artery or vein for which the role and feasibility of surgery are often questioned. This systematic review aimed to identify and evaluate the management strategies used for these complex SI-NETs. The identified studies showed positive outcomes with surgery and multimodality therapy.

## 1. Introduction

Despite the small intestine constituting the vast majority of the gastrointestinal system in length and surface area, neoplasms affecting the small bowel are exceedingly rare. Global incidence is between 0.3 and 2.0 per 100,000, while malignancies of the small intestine represent only 0.4% of all cancers and 2% of malignancies of the gastrointestinal tract [1,2]. The incidence of these tumours is increasing markedly, with a fourfold increase since 1973 in the United States [3,4].

Of the malignant tumours of the small intestine, neuroendocrine tumours constitute approximately 40% and are the most common small bowel tumours in most Western populations [3,5,6].

Small intestinal neuroendocrine tumours (SI-NETs) are derived from the neuroendocrine cells lining the bowel that secrete hormones such as serotonin and digestive enzymes. Over 70% of SI-NETs originate in the ileum, 22% in the duodenum and the remainder in the jejunum [7,8].

Jejunal and ileal NETs rarely secrete hormones and are typically asymptomatic, with occult bleeding and obstruction occurring at a later stage. Consequently, they are frequently diagnosed at an advanced stage. Classic carcinoid syndrome, with flushing, diarrhoea, bronchospasm and carcinoid heart disease, is uncommon, occurring in only 20% and occurs at a late stage once liver metastases are established [9,10].

Distal SI-NETs (diSI-NETs) are typically >2 cm in size at the time of diagnosis, while more than 75% of duodenal NETs are smaller than this, further distal tumours are commonly multifocal with up to 44% having further tumours along the small bowel [11,12]. A total of 80% of diSI-NETs have regional mesenteric lymph node metastases at presentation [13,14], whilst further metastases to the peritoneum and liver are present in 20 and 60%, respectively [15].

Despite the extensive degree of metastasis at presentation, surgical resection of the primary tumour and regional lymph nodes is still recommended for SI-NETs and is associated with improved overall survival and symptomatic control, even in a palliative capacity [14,16].

The small bowel mesentery represents one of the most dominant sites of tumour spread and mesenteric nodal metastases are a hallmark feature of SI-NETs. The mesenteric mass induces extensive fibrosis and desmoplastic reaction in the surrounding mesentery, which, in turn, can lead to bowel obstruction and ischemia [17,18,19]. The mesenteric disease can further extend towards the mesenteric root and encase the superior mesenteric veins and arteries posing unique surgical challenges [14,17,20].

Symptoms related to mesenteric involvement include mesenteric angina, recurrent ascites and lower gastrointestinal bleeding secondary to ectopic small intestinal varices.

The extent of mesenteric disease (Table 1) is described according to the Lardière-Deguelte classification: Stage I describes nodes adjacent to the small bowel; Stage II disease involves distal branches of the SMA near their origin; Stage III involves the trunk of the SMA without the involvement of the first jejunal arteries; this stage is further divided into ‘up’ and ‘down’, when there are less than or greater than 3–4 free jejunal branches, respectively; and Stage IV disease involves the trunk of the SMA and first jejunal arteries [21].

The Ohrvall classification is similar and is now recommended by the North American Neuroendocrine Society (NANETS): Stage I tumours close to the small intestine; Stage II tumours close to the origin of the SMA; Stage III tumours extending along, but not encircling the SMA; and Stage IV tumours describe tumours that extend retroperitoneally, may involve the pancreas, involve the first jejunal branches, and encircle the SMA [22]. 

Standard portal venous phase CT imaging of the chest, abdomen and pelvis is typically the first line of investigation to stage these tumours; however, it has a limited sensitivity of as low as 32% but as high as 82% with the presence of mesenteric disease. Arterial phase imaging may also increase the detection of these hypervascular tumours and in mesenteric disease allows assessment of the relationship of the tumour to the vasculature and adequacy of perfusion to the bowel. CT enteroclysis further improves sensitivity to 87%. Ga-68 PET-CT provides the highest sensitivity for diagnosis of SI-NET at 92–100%. A combination of these modalities is therefore needed for accurate localisation and pre-operative planning in SI-NETs [17,23].

The gold standard surgical approach for SI-NETS with locoregional disease is segmental resection of the affected small bowel coupled with the resection of the regional lymph nodes up to the segmental branches of the superior mesenteric artery and vein [14]. In advanced SI-NETS, removal of mesenteric masses is associated with improved tumour-related symptoms and survival time even in the presence of liver metastasis [14]. However, SI-NETs with significant involvement of the proximal mesenteric root have usually been deemed inoperable over fear of endangering the blood supply to the bowel and are managed primarily with medical treatment [14].

EVOTE is a novel hybrid surgical approach (further detailed below) described by Horwitz et al., entailing pre-operative angiography and embolization to facilitate the complete excision of metastases involving the proximal mesenteric root. SMA angiography is used to identify the involved SMA segment and to look for adequate arterial collateralization and monitor for the development of acute abdominal pain subsequent to balloon occlusion. A mass would be considered unresectable if there was inadequate collateralization. When both adequate collaterals were present and no abdominal pain occurred following occlusion, then an embolization plug was deployed. If abdominal pain occurred, resection could still be attempted as long as good collaterals were present, but no embolectomy would be performed [24].

Though the survival of patients with advanced SI-NETs has been enhanced in recent years with the advent of targeted treatment options such as somatostatin analogues, everolimus, and peptide receptor radionuclide therapy (PRRT) with ^177^Lu-DOTATATE, treatment options for patients with proximal vascular involvement secondary to mesenteric nodal metastases have remained limited [17,20,25,26,27].

Increasingly, more invasive radical and cytoreductive strategies are being trialled to manage this challenging disease. This review aims to summarise these novel surgical techniques in treating SI-NETs with mesenteric nodal metastasis with vascular involvement. 

## 2. Materials and Methods

A systematic literature search with narrative synthesis on studies reporting surgical management of SI-NETs with superior mesenteric artery (SMA) or superior mesenteric vein (SMV) involvement was conducted.

The systematic review followed the recommendations of the Preferred Reporting Items for Systematic Reviews and Meta-Analyses (PRISMA). The protocol has not been registered.

### 2.1. Search Strategy

MEDLINE and Embase databases from 1 January 1970 to 14 January 2023 were searched. The following search terms were used: “superior mesenteric”, “neuroendocrine tumour”, “carcinoid”, “encas*”, “mesen*”, “mesenteric root”. 

### 2.2. Eligibility Criteria

Original papers reviewing and reporting clinical outcomes for treatment of SI-NETs with SMA/SMV encasement, involvement, or occlusion, as demonstrated by pre-operative imaging or findings during surgery were included. Only English language studies were included. Non-human studies, evaluating NETs outside the small bowel, involving patients with severe co-morbidities or studies reporting outcomes of palliative intestinal bypass procedures were excluded (Table 2).

### 2.3. Study Selection

Two authors (EK and SL) independently performed literature searches and determined eligibility of studies. Consensus on final eligibility was reached through discussion between the authors.

### 2.4. Data Extraction and Synthesis

The following data were extracted from included studies: first author’s name, month and year of publication, study design, number of patients, type of SMA/SMV involvement, and method of SI-NET treatment intervention. Outcomes including length of follow-up, median survival time, actuarial survival and recurrence and improvement in quality of life were also collected.

Narrative synthesis and discussion of their results are presented. Meta-analysis was not performed due to the small number of studies and clinical heterogeneity.

## 3. Results

**Figure 1:**
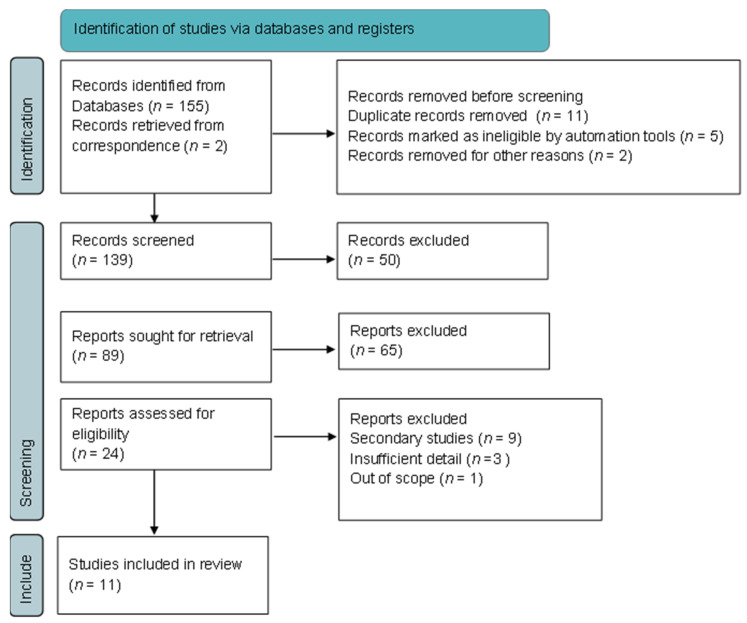
Flowchart of inclusion and exclusion of screened studies.

A total of 155 papers were identified from the database search and, after screening, 11 studies met the eligibility criteria and were included in the review (Figure 1).

These 11 studies were all non-randomised cohort studies reporting outcome data on 279 patients with SI-NETs and SMA/SMV encasement, occlusion or involvement at the mesenteric vessel root (Table 3). The treatment strategies described include (1) abdominal debulking and resections [22,28,29,30,31,32,33], (2) endovascular occlusion and tumour excision (EVOTE) procedure [24], (3) auto-transplantation [34] and (4) endovascular stenting of the SMV [19,35].

### 3.1. Abdominal Debulking and Resection

Five studies were found on debulking and resection of mesenteric root masses. These have shown symptom improvement [22,29,30,31] and improvement in survival [28].

Ohrvall et al. report outcomes from 56 patients with SI-NET who underwent laparotomy with dissection of mesenteric masses. From this study, they developed their own classification of SI-NET mesenteric disease described above and adopted by NANETS (see Table 1).

A total of 9 (16.1%) of these patients had Stage IV mesenteric disease as per their classification. SI-NETs with Stage I and II mesenteric masses were successfully excised with locoregional excision, with Stage II disease additionally requiring concomitant right hemi-colectomy. Stage III tumours, although initially appearing unresectable, were successfully dissected free from the mesenteric artery followed by small bowel resection and right hemi-colectomy. The Stage IV tumours were not resected, but transected, to allow resection of the ischaemic or obstructed bowel. All patients had primary anastomoses formed. Post-operatively patients reported improvement in pre-operative symptoms (abdominal pain, weight loss, diarrhoea and intestinal obstruction). Non-fatal complications requiring re-operation occurred in five patients (fluid collection/hematoma, *n* = 2; adhesional intestinal obstruction, *n* = 3), and one patient died 4 weeks post-operatively from anastomotic leak. Long-term survival data are not presented in this study [22].

Bertani et al. performed a single-centre retrospective study of 49 patients with SI-NET and mesenteric disease; 36 (75.5%) of these patients also had distant metastases. All of these patients underwent laparotomy with an intended resection of the primary site and mesenteric disease, 37 underwent resection, whilst 12 (24.5%) were unresectable [28].

All patients classified as SMA I (*n* = 13) underwent locoregional resection, while 88.8% of the SMA II group (*n* = 9) and 78.9% of the SMA III down group (*n* = 19) were resected. However only one case classified as SMA III up (*n* = 7) was resectable, and no patients in the SMA 4 group (*n* = 2) underwent resection. [36] No cases with proximal SMV infiltration (*n* = 5) were resected, while 92% of patients with no SMV involvement (*n* = 25) and 73.7% of distal SMV infiltration (*n* = 19) underwent resections [28]. The median overall survival for unresectable patients was 90 months, while the median was not reached during follow-up for the resected patient (*p* = 0.004). The 8-year survival was 81% for the resected patients and 40% for the unresectable patients.

Boudreaux et al. reported on patients with advanced SI-NETs undergoing palliative intent resections. In their retrospective review of 86 cases, they identified 12 with mesenteric vascular encasement, although the exact level at which this was observed is not noted. Complete tumour excision via debulking was possible in 83% (*n* = 10) of patients relieving intestinal ischemia and mean pre-operative and post-operative Karnofsky performance scores were 65 and 85, respectively (*p* < 0.0001), indicating an improved post-operative health-related quality of life (HRQOL). While morbidity and mortality rates for this particular subset of SBNET patients are not specified, the overall post-operative 30-day mortality rate was 0% and overall survival was 66% at a mean follow-up of 22.8 months (range 3–72 months) [29].

Improved post-operative Karnofsky physical performance scores were also shown by Gulec et al. in their retrospective study of 30 patients with advanced SI-NET. In their study, successful removal of the mesenteric tumour was possible in three out of the five patients (60%) who presented with encasement of the mesenteric vessels. The exact level at which encasement occurred is not reported. The mean Karnofsky physical performance scores improved following surgery (55 pre-operatively and 85 post-operatively, *p* < 0.02) across the total sample size, but there are no data specific to those who presented with mesenteric encasement. Three deaths were reported overall due to disease progression; however, it is unclear if these occurred in patients with SMA/SMV encasement [30].

Makridis et al. described their experience of patients with advanced SI-NET who underwent surgery (*n* = 51) and the majority (86%) of those patients had some degree of mesenteric metastases [31]. Although the degree of involvement of the SMA/SMV is not explicitly described in this sample, it is mentioned that 31 patients had significant growth in the mesentery, often extending to the origin of the mesenteric vessels. Of these, 6 cases underwent only exploratory laparotomy or palliative procedures, while 25 cases were subjected to debulking procedures leaving the portions of the mass that surrounded the trunk of the SMA/SMV due to concerns regarding compromising vascular supply to the bowel. Post-operatively there was complete resolution of intestinal obstruction or ischaemia in those who suffered from this pre-operatively and improvement of abdominal pain and diarrhoea. However, outcomes are presented for the aggregate sample and not just for the patients with SMA/SMV involvement, making it difficult to assess the results and complications for this subset of SI-NET patients. Severe complications included 13 deaths, all from progressive carcinoid disease except for two instances: one death from post-operatively bleeding, and one death in a patient who developed short-bowel syndrome after re-operation for ileus requiring resection [31].

Lastly, reports by Kasai [32] and Wonn [33] suggest that, contrary to the above studies, it is the presence of liver metastasis over resection of mesenteric nodal metastases that dictates post-operative survival.

Kasai studied 106 patients who underwent resection of SI-NETs; 66 had large mesenteric metastases >2 cm in size and of these 15 underwent complete resection, whilst 20 underwent incomplete resection. Overall and progression-free survival were not significantly different between those who had complete versus incomplete mesenteric mass resection. The absence of large mesenteric metastasis was significantly associated with an increase in 5-year overall survival to 92.6% from 65.5% in those with mesenteric masses who underwent complete resection (*p* = 0.018). These were also significant on multivariable analysis for liver involvement >25% (hazard ratio 3.62, 95%CI 1.13–10.0) and large mesenteric metastasis (hazard ratio 4.69, 95% CI 1.63–17.6) in predicting 5-year overall survival.

Wonn and colleagues studied 272 patients with a resection or curative attempt resection of SI-NET primaries and mesenteric metastases; 98 patients (89%) had complete resection, 10 (5%) had incomplete resection and 14 (6%) were deemed unresectable due to encasement of the mesenteric vessels. Nodal status was not associated with overall survival on multivariable analysis (Hazard ratio 1.11, 0.38–3.28) whilst liver metastasis was (hazard ratio 5.05, 95% CI 1.2–1.3).

### 3.2. Auto-transplantation

Kitchens et al. described a challenging case of SI-NET at the root of the mesentery encasing the SMV, occluding multiple branches of the SMA including the ileocolic and first jejunal branches and duodenum and head of pancreas. The tumour was also noted to involve the inferior vena cava and aorta.

Laparotomy was performed with Kocherisation of the duodenum to expose the great vessels and head of the pancreas. Lymphadenectomy was performed along the aorta and inferior vena cava. A classical Whipple’s procedure was then performed with the removal of the pylorus, head of the pancreas, duodenum and distal common bile duct.

The small bowel was then resected in two segments, an ileocaecal segment (the right colon and the transverse colon were also resected) and an ileojejunal segment. This was to allow separate vascular anastomoses from the ileocolic and jejunal vessels respectively.

The bowel segments were placed on a bench with ice and with University of Wisconsin preservation solution.

The SMV and SMA were then divided leaving 2 cm stumps. The SMA and SMV stumps were anastomosed to the ileojejunal segment while end-to-side anastomoses were formed to the aorta and infrarenal vena cava from the jejunal segment. Choledocho-, pancreatico- and gastrojejunostomies were performed to restore continuity. The two small bowel segments were also anastomosed. The caecum was brought out as a caecostomy, while the descending colon was left stapled off initially.

The patient later developed thrombosis of the SMA, which required relaparotomy thrombectomy and heparinisation. Ultimately the ileojejunal segment became necrotic and required excision with refashioning of the hepatobiliary and gastric anastomoses to the ileocaecal segment and refashioning of the vascular anastomoses to this segment. The large bowel was ultimately anastomosed also.

The patient ultimately had 70 cm of ileum left and required TPN post-operatively. Despite being a palliative intent procedure, the authors reported a complete excision. The patient survived 2.5 years post-operatively with no evidence of recurrence and stopped TPN [34].

### 3.3. EVOTE

Horwitz et al. demonstrated that complete elimination of the tumour mass by EVOTE (*n* = 14 procedures attempted, in 13 participants) was successful in 86% of cases (*n* = 12) with SI-NETs encasing the SMA, as indicated on CT angiography [27]. Participants deemed eligible for EVOTE had Stage II or Stage III nodal disease as per the classification by Lardière-Deguelte [21]. Two patients had incomplete tumour excision: one was inoperable due to proximal SMV involvement and the other the tumour was incompletely resected from the duodenum and proximal SMA. While the 30-day mortality rate was 0%, local recurrence was seen in one patient at 31.8 months post-operatively, who subsequently underwent a second EVOTE procedure, yielding a complete excision. Four patients experienced complications, including a prolonged post-operative ileus, which was managed conservatively, and a chyle leak that resolved with conservative management. Half of the complications (*n* = 2) were severe, comprising small bowel anastomotic leaks, in which one patient required re-operation [24].

### 3.4. Stenting

SI-NETs with involvement of the mesenteric root can cause occlusion of blood flow through the mesenteric vessels and the thin-walled veins of the portal system are especially at risk of occlusion. In cases where the SMV is involved, stenting can be attempted to normalise venous blood flow being compromised by the mass. In brief, this involves inserting a self-expandable metallic stent trans-hepatically through the portal vein and placing it in the upper portion of the SMV [19]. While this may be considered a stand-alone palliative procedure, it has the potential for use in conjunction with other interventions with curative intent.

Hellman et al. reported outcomes from seven patients with inoperable SI-NETs presenting with encasement of the main branches of the superior mesenteric vein and artery [19]. Pre-intervention CT scans demonstrated signs of venous stasis in affected portions of the small intestine in all cases, including the following radiological observations: thickening of the intestinal wall with signs of oedema, development of tortuous collateral veins, and obstruction of venous blood flow through the superior mesenteric vein. As such, these seven patients were selected to undergo SMV stenting as a means of easing venous compromise and relieving symptoms. The authors report up to 80% symptom resolution in four out of the seven included patients (57%), as determined by pain scores on a visual analogue scale, decreased diarrhoea, and subjective wellness reports. In the successful group, symptom improvement was associated with the degree of normalization of blood flow through the SMV. Of these four patients, one had experienced ascites from the chyloperitoneum, and this completely resolved after SMV stenting. The remaining three participants either had no improvement (*n* = 2) or worsening of symptoms, though this deterioration was unrelated to stenting (*n* = 1). In terms of post-procedure complications, one patient developed bleeding from the hepatic puncture site, and this was managed with radiological embolization. No other significant complications were reported by the authors [19].

### 3.5. Stenting versus Debulking

Daskalakis et al. conducted a retrospective analysis on patients with advanced SI-NETs (*n* = 528), of whom 20 experienced impaired intestinal circulation due to large mesenteric masses and associated desmoplastic reactions in the mesenteric root, compressing the SMA/SMV [35].

Of these, 12 patients underwent stenting, and the remaining 8 patients underwent laparotomy and locoregional resection with a view to decompressing the SMV by mesenteric dissection. The level of involvement of the mesentery is described as encompassing the mesenteric root at the level of, or above, the first part of the duodenum and encasing the superior mesenteric vessels. Subjective symptom alleviation was yielded in 25% of stented patients and 62.5% of laparotomy patients as assessed by patient charts. Eight patients in the stenting group had radiological follow-up, confirming the normalization of blood flow in the SMV. Of note, there was no significant difference observed in the 30-day mortality between patients who underwent stenting compared to resection. However, there was a lower rate of morbidity after stenting (*p* = 0.036) and hospital stays were shorter (3 versus 9 days; *p* = 0.005) in these patients compared to the laparotomy patients. The median survival time of stented patients was 1.6 times greater than that of patients who underwent resections, but this was not statistically significant (*p* = 0.81) [35]. As with the findings reported by Hellman et al. [19], the patients who experienced ascites pre-stenting (*n* = 3), developed regression of ascites post-stenting [35].

## 4. Discussion

Small intestinal neuroendocrine tumours with mesenteric vascular involvement represent a difficult entity to treat due to concerns regarding midgut circulatory compromise and resultant short gut syndrome. To the authors’ knowledge, this is the first systematic review to address this subject, and the evidence presented here suggests that mesenteric root involvement, and even encasement, should not be considered an absolute contraindication to surgical exploration. Rather, a more nuanced approach, taking into account the degree of involvement of the mesenteric vessels and considering different surgical and invasive approaches should be considered.

The EVOTE approach showed promise in further delineating which mesenteric masses merit an endeavour at resection. It was attempted in patients with Stage II or III nodal disease, graded according to the classification systems postulated by Ohrvall and colleagues [22]. Of the 16 patients referred from other centres as unresectable, 81% were deemed candidates for the EVOTE procedure with an 86% success rate for complete resection of the mesenteric mass. The embolectomy plug conferred the benefit of facilitating intraoperative identification of the feeding SMA tributary by manual palpation, allowing it to be controlled, and enabling mesenteric mass dissection. While the EVOTE procedure had a high efficacy rate, it also suffered from a 14% anastomotic leak rate that the authors attribute to underappreciated ischemia in one case, and venous insufficiency in the other, which had unresectable SMV involvement. However, the study had a relatively small sample size and further evaluation is required to properly assess anastomotic outcomes following the EVOTE procedure [27].

It is noteworthy that in all the studies that involved resection or debulking, there was only one recorded instance of short-bowel syndrome, which occurred during a small bowel resection for post-operative ileus, and it is unclear if the patient in question had a mesenteric mass debulked as the authors presented aggregate morbidity data for all their SI-NET patients [31]. This indicates that in appropriately selected cases, a safe resection of masses involving the SMA/SMV is possible without causing significant ischaemia to the gut. Furthermore, current NANET guidelines suggest referral to specialist NET centres who manage higher volumes of complex SI-NETs, to aid in the assessment of resectability of these technically challenging mesenteric masses [14]. The latter can be especially important when there is a concern for the development of vascular compromise and/or short gut syndrome. In support of this, Boudreaux and colleagues were able to successfully resect 6 out of 12 encasing mesenteric masses at their institution that were previously felt to be unresectable when surgically explored at the referring centres [29]. Similarly, when Horowitz et al. re-evaluated mesenteric tumours previously deemed inoperable, they were able to achieve total resection of the mesenteric mass in 69% of those referred as unresectable with the EVOTE procedure at their site [27].

In cases with very proximal mesenteric root involvement, intestinal auto-transplantation may be a potential alternative to resection or debulking. Intestinal auto-transplantation has been employed in relation to a variety of abdominal tumours involving encasement of the mesenteric vessels, allowing complete resection of tumours involving the mesenteric root and early independence from TPN [34,37,38]. However, there are considerable operative risks associated with this approach with complications such as post-operative haemorrhage, SMA thrombosis leading to intestinal autograft failure, early tumour recurrence (especially in pancreatic ductal tumours), and pancreatic leaks being reported in the literature. While Kitchens et al. successfully used auto-transplantation to resect a mesenteric mass that was encasing the SMV and extending along the mesenteric root, the procedure was complicated by SMA thrombosis and pancreatic leak [34]. Ultimately the procedure was a success, but the patient required a re-operation and further resection of the bowel. Further studies are needed to better explore the role and operative risks associated with this procedure for advanced SI-NETs.

In patients with proximal SMV involvement in which resection is felt to be technically not feasible, stenting the SMV has shown some positive results. Studies by Hellman et al. and Daskalakis et al. demonstrate that in carefully selected patients, insertion of a self-expandable stent through a stenotic superior mesenteric vein may improve abdominal symptoms in those patients with SI-NETs with superior mesenteric involvement [19,35]. Success for this procedure ranged from 25 to 57% and was primarily assessed based on symptom relief [19,27]. Furthermore, stenting was seen to be particularly useful in patients with ascites from chyloperitoneum related to SMV involvement, with stenting being associated with regression of the ascites [19,35]. Success with stenting is likely operator-dependent, contingent on appropriate stent placement, as well as on the selection of appropriate patients where SMV stenosis is present. It is important to note that the study by Daskalakis and colleagues included patients with SBNETS and SMA/SMV involvement stemming from fibrotic reactions, due to either adjacent mesenteric masses or para-aortic lymph node metastases, and these were not dealt with separately in the paper. Although seen as a palliative procedure used in inoperable patients, Daskalakis et al. argue that stenting can also be considered as a bridging therapy, perhaps in conjunction with targeted medical therapy, for patients previously deemed to have inoperable disease [35]. More robust studies are needed to further investigate this potential. Hybrid surgical and interventional radiological approaches, however, show great promise in the management of challenging diseases involving the mesenteric vessels.

The standardization of the assessment of the level of mesenteric involvement via classification systems is tantamount in operative planning for these complex SI-NETs and offers a more objective assessment of resectability. The classification system developed by Ohrvall and colleagues is now widely accepted and referenced in the NANETS consensus guidelines for the management of SI-NETs with mesenteric involvement. According to this classification, mesenteric tumours up to Stage III (involving the SMA near the trunk but not encircling it) are potentially resectable. For Stage III disease, the recommendation is to track and dissect the lymph nodes proximally along the superior mesenteric vascular branches and selectively excise these, though, in practice, this can be technically challenging in cases associated with significant mesenteric desmoplasia. Stage IV tumours (tumours that encircle the SMA and involve the origin of the proximal jejunal arteries, or extend retroperitoneally behind or above the pancreas) are not deemed amenable to resection [22]. For Stage IV tumours, Ohrvall and colleagues suggest transecting the mass in situations where there is a compromised bowel (ischaemic or obstructed) [22]. Similarly, using the Lardière-Deguelte classification based on pre-operative CT scans, Bertani et al. found that resection of SI-NETs was possible in patients with more distal SMA/SMV involvement. However, in the presence of proximal SMA involvement (less than four disease-free proximal branches), proximal SMV involvement or the presence of mesenteric fibrosis, resection was less likely to be fruitful [28].

The European Society for Neuroendocrine Tumours (ENETS) guidelines are more conservative in their recommendations for surgical management of SI-NETs with mesenteric disease. SI-NETs with mesenteric deposits “without major involvement of the mesenteric vessel root and/or retroperitoneum” are deemed borderline resectable. Mesenteric deposits surrounding the vessel roots or extending into the retroperitoneum are considered unresectable in the same way as Ohrvall Stage IV disease as in the NANETs guidelines [39,40].

The limitations of current evidence regarding surgical interventions for SBNETs with mesenteric root involvement include the lack of randomised control trials, limited sample sizes of current studies and predominant reliance on retrospective studies, likely influenced by the fact that SBNETS are still relatively rare. Furthermore, there is a lack of a standardised approach in describing the level of involvement of the mesenteric vessels with some of the studies reviewed mentioning encasement of mesenteric vasculature without specifying the level at which this encasement was found [29,30,31]. This makes it difficult to draw firm conclusions about the possibility of debulking/excising more proximal SMA/SMV masses. Larger scale prospectively designed studies are needed to further evaluate the different invasive and surgical techniques currently available and summarised herein. However, data in the present review do offer some interesting treatment avenues for SI-NETS with mesenteric masses previously felt to be unresectable, as well as highlighting the importance of a standardised approach to the assessment of resectability of these most challenging of tumours.

## 5. Conclusions

In this review, we have identified several management options for the treatment of small intestinal neuroendocrine tumours involving the mesenteric vasculature. At present, the literature consists predominantly of level IV evidence showing the safety of these approaches, with no prospective or randomised studies identified. For Ohrvall Stage I and II tumours, it is established that surgical resection is feasible and associated with an improved overall survival, although published data are limited.

Future research should comprise appropriately powered randomised controlled trials evaluating these novel interventions versus standard of care, in particular, for Ohrvall Stage III and IV disease, where controversy around resectability and the oncological benefit of surgical resection remains.

## Figures and Tables

**Table 1:** Classification systems of SI-NETs involving the mesentery; SMA—superior mesenteric artery.

	Classification	
Stage	Ohrvall	Lardière-Deguelte
I	Close to small intestine	Adjacent to small bowel
II	Close to origin of SMA	Distal branches of SMA near origin
III	Along but not encircling SMA	Involve trunk of SMA without involvement of first jejunal arteries Up: >3 to 4 free jejunal branches Down: <3 to 4 free jejunal branches
IV	Extending retroperitoneally, may involve pancreas, involve first jejunal branches and encircle SMA	Involving trunk of SMA and first jejunal arteries

**Table 2:** Inclusion and exclusion criteria for systematic review.

Criteria	Inclusion	Exclusion
Databases	Medline and Embase	-
Time period	1 January 1970 to 14 January 2023	-
Language	English	Non-English language
Publication type	Primary observational or interventional study	Review article, case report, abstract only
Population	Humans	Animals, laboratory studies
	Studies involving treatment of SI-NETs with SMA/SMV encasement, involvement, or occlusion demonstrated by pre-operative imaging or findings during surgery	Alternative pathology, NET with alternative primary site/ unknown primary
Interventions	Surgical resection of SI-NET with SMA/SMV involvement with curative intent	Palliative bypass or medical therapy only

**Table 3:** Summary of included studies.

Study	Sple Size (N)	Stage	Urgency of Treatment	Surgical Treatment	Mean Follow-Up (Months)	Median Survival After Intervention (Months)	Total Deaths	Local Recurrence
Hellman et al. [19]	(314 total) 7 palliative bypass	34 liver mets Level of mesenteric masses not stated	121 (46%) Emergency Surgery	Stenting	36		2 (29%)	0
Makridis et al. [31]	121 patients with SI-NET overall 51 with mesenteric disease	62% Liver metastases Level of mesenteric metastases not stated	23 elective 44 emergency (overall)	Resection *n* = 20 of those 13 had minor mesenteric mets Debulking (*n* = 25) Exploratory laparotomy or palliative intestinal bypass (*n* = 6)	33.6	Mean: 29 Median: 74.5	13 (25%) In total study	
Kitchens et al. [34]	1	Encasing mesenteric vein and extending to mesenteric root and pancreas	Elective	Auto-transplantation Debulking	30	36+ (still alive)	0	0
Boudreaux et al. [29]	82	Liver metastases in 79% Mesenteric vascular encasement 14.6%	Not stated	Debulking	22.8			
Gulec et al. [30]	30	Liver metastases in 93% Mesenteric vessel involvement 17%	Not stated	Debulking	11			
Horwitz et al. [24]	13	All Stage II or III as per Ohrvall classification	All elective	EVOTE	15.1		0	1 (8%)
Ohrvall et al. [22]	56	24% Stage I 22% Stage II 38% Stage III 16% Stage IV	All elective	Resection			1 (2%)	
Daskalakis et al. [35]	20 Total = 36 16 patients had fibrosis causing obstructive uropathy	100% Liver metastases Stage not stated	Not stated	Stenting (*n* = 12) Resection (*n* = 8)	120	28 (Stent) 17.5 (Resection)	2 (10%) (1 from each)	
Bertani et al. [28]	49	26.5% Stage I 18.4% Stage II 38.8% Stage III down 12.2% Stage III up 4.1% Stage IV	All elective	Resection (*n* = 1) of SMA encasing group (*n* = 0) of SMV encasing group	68			
Kasai et al. [32]	106	Liver metastasis: 62% for >2 cm mesenteric masses; 53% for <2 cm mesenteric masses Level of mass not stated	Not stated	Resection	46.9		25 (23.5%)	
Wonn et al. [33]	300 (272 underwent resection of SI-NET and mesenteric mass)	81% Liver metastases All patients had peritoneal carcinomatosis Level of mesenteric mass not stated	All elective	Resection	62	Not reached	64 (23.5%)	

## Data Availability

No new data were created or analyzed in this study. Data sharing is not applicable to this article.
